# Porcine Organotypic Epicardial Slice Protocol: A Tool for the Study of Epicardium in Cardiovascular Research

**DOI:** 10.3389/fcvm.2022.920013

**Published:** 2022-07-18

**Authors:** Davide Maselli, Rolando S. Matos, Robert D. Johnson, Davide Martella, Valeria Caprettini, Ciro Chiappini, Patrizia Camelliti, Paola Campagnolo

**Affiliations:** ^1^Cardiovascular Section, Department of Biochemical Sciences, University of Surrey, Guildford, United Kingdom; ^2^London Centre for Nanotechnology, King's College London, London, United Kingdom; ^3^Centre for Craniofacial and Regenerative Biology, King's College London, London, United Kingdom

**Keywords:** epicardium, *ex vivo* model, organotypic culture model, regenerative medicine, myocardial infarction

## Abstract

The epicardium has recently gained interest in the cardiovascular field due to its capacity to support heart regeneration after ischemic injury. Models to study the epicardium of large animals *in vitro* are limited and mainly based on epicardial cell isolation/differentiation from stem cells, followed by 2D cells culture. In this method paper, we describe the procedure to obtain and culture 3D organotypic heart slices presenting an intact epicardium, as a novel model to study the epicardial physiology and activation. Epicardial slices are obtained from porcine hearts using a high-precision vibratome and retain a healthy epicardial layer embedded in its native extracellular environment and connected with other cardiac cells (cardiomyocytes, fibroblasts, vascular cells etc.). Epicardial slices can be cultured for 72 h, providing an ideal model for studying the epicardium physiology or perform pharmacological interventions/gene therapy approaches. We also report on methods to assesses the viability and composition of the epicardial slices, and evaluate their architecture in 3D through tissue decoloration. Finally, we present a potential application for a nanomaterial-based gene transfer method for tracking of epicardial cells within the slice. Crucially, given the similarity in morphology and physiology of porcine heart with its human counterpart, our system provides a platform for translational research while providing a clinically relevant and ethical alternative to the use of small animals in this type of research.

## Introduction

After myocardial injury, adult epicardial cells transiently re-express embryonic genes and actively participate to cardiac repair both through direct differentiation into cardiac cells and by producing reparative paracrine factors (1, 2). Recent discoveries of the role of the epicardium in heart repair have generated interest in developing strategies to exploit its regenerative potential for patient therapy (3–5). Due to the biological complexity of the cardiovascular system, the choice of the animal model is critical owing to significant inter-species differences (6, 7). Currently, studies on the epicardium are based either on 2D *in vitro* culture systems or on fate-tracking animal models, such as zebrafish and mice. *In vitro* experiments conducted with stem cell-derived epicardial cell lines, immortalized cell lines or primary isolated epicardial cells, provided a comprehensive knowledge of the secretome and transcriptional profiling of the epicardium (4, 8–12). *In vivo*, zebrafish represent an interesting model due to its remarkable heart regeneration capacity, which is dependent on epicardial signals and epicardial cell trans-differentiation (13–16). Zebrafish studies have provided valuable mechanistic insights into the regenerative process of the heart and specifically the epicardial contribution (16). Mouse models are extensively utilized for their amenability to genetic modification, which allows the study of single gene function *in vivo* and fate mapping studies. These animal models were crucial in the identification of the pivotal role of the transcription factors WT1, Tbx18 and TCF21 in guiding the epicardium development and driving its repair potential (2, 17–19). However, for research aimed at clinical translation, it is essential that results from small vertebrates' studies are confirmed in a large animal models that more closely resemble the human heart physiology (6, 19).

Cardiac slices are well-established *ex vivo* models consisting of thin tissue slices obtained from the cardiac muscle (20, 21), and have been used in the past two decades for electrophysiological and pharmacological drug testing (21, 22). In our recent paper we described for the first time the preparation and characterization of heart slices derived from the epicardial portion of the heart, and their use for the investigation of this intriguing reservoir of regenerative cells (23). Our epicardial slices encompass the epicardial/myocardial interface while also preserving the cellular and extracellular structure of the cardiac tissue. Here we aim at producing an extensive and detailed protocol to support interested user in the production of epicardial slices, and their culture.

In part, this is due to technical difficulties as the epicardium is normally sacrificed to attach the specimen to the vibratome holder in order to ensure the alignment of the myocardial fibers within the tissue during cutting (24, 25). In this protocol, we modified previously developed tissue embedding (26, 27) and cutting (28, 29) procedure by flattening the tissue against a compliant surface during the embedding and then cutting the tissue starting from the epicardial side. This protocol exclusively enables to preserve the epicardial layer and obtain both healthy epicardial slices and functional cardiac slices. Epicardial slices contain both the epicardium and the myocardium and are amenable to culture for up to 72 h, maintaining vitality and morphology of the epicardial cells. They provide a suitable platform for multiplexing assays for drug/gene therapy discovery focused on the epicardium, and to study its interaction with the myocardial tissue, in a tunable culture system.

Importantly, epicardial slices have the potential to replace and reduce the use of small animals for research on the epicardium. Our slices were successfully derived from both experimental and abattoir porcine tissues, and with an adult heart yielding to 15–25 slices, each providing one experimental sample. This enables the multiplexing of assays, improving consistency and helping reducing the number of small animals used in cardiovascular research.

In summary, in this method paper we described in detail a robust and efficient protocol for obtaining and culturing epicardial slices, that can be used to study the physiological and reparative roles of the epicardium in an easy and cost-effective manner. Our system is based on large animal tissue, which closely resembles the human heart and has the potential to substitute or reduce the use of small animals in this type of research.

## Materials and Equipment

### Tissue Samples

Four to Six weeks old piglet hearts were obtained from The Pirbright Institute, UK. Animal procedures were carried out under the Home Office Animals (Scientific Procedures) Act (1986) and approved by the Animal Welfare and Ethical Review Board (AWERB) of The Pirbright Institute. The animals were housed in accordance with the Code of Practice for the Housing and Care of Animals Bred. All procedures were conducted by Personal License holders who were trained and competent and under the Project License PPL70/8852. Sixteen to twenty weeks old pig heart were obtained from Newman's Abattoir (Farnborough, UK).

### Preparation of Solutions

Preparation of cardioplegia solution: This solution is required for the perfusion of the piglets' hearts and the transportation of cardiac tissue. To prepare 1 liter of solution, add the following: 6.43 g of sodium chloride (final concentration: 110 mM), 1.19 g of potassium chloride (final concentration: 16 mM), 3.25 g of magnesium chloride (final concentration: 16 mM) and 0.13 g of calcium chloride (final concentration: 1.2 mM), in 800 ml of double-distilled water (ddH_2_O). Once all the reagents are dissolved, measure and adjust the pH to 7.4 with 10 mM sodium bicarbonate solution and then add ddH_2_O to reach final volume (1 L). Cool down the solution to 4°C (see info in Table 1).

**Table 1:** Components of solution.

**Cardioplegia**			**Normal Tyrode's solution**			**Normal Tyrode's solution for recording**		
**Composition**	**Manufacturer**	**Concentration (mM)**	**Composition**	**Manufacturer**	**Concentration (mM)**	**Composition**	**Manufacturer**	**Concentration (mM)**
NaCl	VWR Chemicals	110	NaCl	VWR Chemicals	140	NaCl	VWR Chemicals	140
CaCl2	Sigma-Aldrich	1.2	CaCl2	Sigma-Aldrich	1.8	CaCl2	Sigma-Aldrich	1.8
KCl	Sigma-Aldrich	16	KCl	Sigma-Aldrich	6	KCl	Sigma-Aldrich	4.5
MgCl2	VWR Chemicals	16	MgCl2	VWR Chemicals	1	MgCl2	VWR Chemicals	1
			Glucose	Sigma-Aldrich	10	Glucose	Sigma-Aldrich	10
			HEPES	VWR Chemicals	10	HEPES	VWR Chemicals	10
			BDM	Sigma-Aldrich	10			
*add NaHCO3 (10mM) to pH 7.4			*add NaOH (10M) to pH 7.4			*add NaOH (10M) to pH 7.4		

*The component needs to be added to adjust the pH of the solution.

Preparation of Normal Tyrode's (NT) solution: Cardiac tissue is cut in ice cold NT solution with the addition of 2,3-Butanedione monoxime (BDM), an excitation–contraction uncoupler that block cardiomyocytes' beating preventing their damage during slicing. After cutting, the epicardial slices recover for at least 30 min at room temperature in the same solution. To make 1 liter of NT solution, add the following to 800 ml of ddH_2_O: 1.00 g of BDM (final concentration: 10 mM), 4.09 g of sodium chloride (final concentration: 140 mM), 0.45 g of potassium chloride (final concentration: 6 mM), 1.8 g of glucose (final concentration: 10 mM), 2.38 g of HEPES (final concentration: 10 mM), 0.20 g of magnesium chloride (final concentration: 1 mM) and 0.20 g of calcium chloride (final concentration: 1.8 mM). Once all the reagents are dissolved, adjust the pH of the solution to 7.4 by adding drops of 2 M sodium hydroxide solution (Sigma-Aldrich) and then add ddH_2_O to reach final volume (1 L). **Tip**: to improve slice viability, measure and fine tune the pH at the working temperature of the NT solutions (4°C for slicing NT solution and room temperature for recovery NT solution) and check the osmolarity of the NT solution to be in between 290 and 320 mOsm/L (see info in Table 1).

### Equipment and Reagents

Equipment and reagents to obtain epicardial slices are listed separately for each phase of the protocol.

#### Excision of the Heart

- Anatomical forceps, Curved, 10 cm (Graefe)
- Disposable sterile scalpel (Torge Surgical Scalpels -size 21)
- Hermetic plastic box
- Scissors, straight, 16.5cm (Lexer)

#### Heart Perfusion

- 3-way valve (Tro-venoflow 3, Torge)
- Anatomical forceps, curved, 10 cm (Graefe)
- Nylon cable ties 100 × 2.5 mm
- Polycarbonate connector female luer hose barb adapter 1/4" (Masterflex Fitting)
- Polystyrene box with lid
- Scissors, straight, 16.5 cm (Lexer)
- Syringe 100 ml sterile (BD Plastipak Catheter-Tip)

Reagents:

- Cardioplegia solution 1L at 4°C

#### Slicing

- 2 × Petri dishes 92 × 16 mm (Sarstedt)
- 3–4 × Sterile plastic Pasteur pipettes (Agar scientific)
- 3D printed plastic ring (details in Supplementary File 1)
- 6- well Millicell cell culture inserts, pore size 0.4μm (Merck)
- 6-well plates × 3 (Customized with holes in the base of each well, Sarstedt)
- Anatomical forceps, curved, 10 cm (Graefe)
- Disposable sterile scalpel (Torge surgical scalpels -size 21)
- Double edge razor blades (Wilkinson Sword)
- High precision vibratome (Leica, VT1200S)
- Plastic box 30 × 40 × 7cm
- Polystyrene Box 60 × 40 × 40cm
- Self-sealing sterilization pouch (Qualitix)
- Single edge steel blades (Fast Mover Tools)
- Thermometer (RS PRO Digital Thermometer)
- Water bath set at 37°C

Reagents:

- Agarose (Invitrogen UltraPure Agarose)
- Cyanoacrylate glue (Solv-X)
- Low melting agarose (ThermoScientific, TopVision Low Melting Point Agarose)
- NT solution: 0.5 L at 4°C and 1 L at room temperature

#### Culture

- 12- well plates (Sarstedt)
- 3D-printed petri dish insert (Supplementary File 2)
- 8 mm-high Polydimethylsiloxane (PDMS) pillars array
- Anatomical forceps, curved, 10cm (Graefe)
- BioFlo120® control station (Eppendorf)
- CO_2_ incubator (Galaxy® 48R – Eppendorf)
- Entomologist pins (A1 - 0.14 × 10 mm, Watkins & Doncaster)
- Petri dish 92 × 16mm (Sarstedt)
- Self-sealing sterilization pouch (Qualitix)
- Syringe Filter Unit, 0.22 μm, polyethersulfone, 33 mm (Medical Millex-GP, Merck)

Reagents:

- Polydimethylsiloxane (PDMS) (SYLGARD 184)
- 2,3-Butanedione monoxime (BDM) (Sigma-Aldrich)
- Ethanol 70% (AnalaR NORMAPUR)
- NT solution: 50 ml at room temperature
- Medium 199 (Sigma-Aldrich)
- Insulin, transferrin, and sodium selenite (ITS) Liquid Media Supplement (100 ×) (Sigma-Aldrich)
- Penicillin-Streptomycin (Sigma-Aldrich)

## Methods

This protocol uses hearts from animals euthanized for research purposes or human consumption obtained from the Pirbright Institute (Pirbright, UK) or the Newman's Abattoir (Farnborough, Hampshire, UK). Before transportation, adult hearts are dissected and piglets' hearts are perfused. Tissue samples are then transferred to the laboratory (within 20–30 min) submerged in cardioplegia solution to arrest the heart in non-contracted state, preventing tissue damage. Once in the lab, 8 × 8 mm cubes are obtained from the cardiac tissue and embedded in low meting agarose and sliced. Our embedding procedure allows to maintain flat and alive the epicardium during slicing. Tissue slices are then transferred to the recovery bath for at least 30 min before further handling. In the following paragraphs we describe in detail the steps to produce epicardial/myocardial slices for *ex vivo* culture.

### Excision of the Heart

Piglets weighing between 6 and 12 kg are euthanized with an intravenous administration of 10 ml of pentobarbital (Dolethal 200 mg/ml solution for injection, Vetoquinol UK Ltd). After exsanguination, the thorax is opened with a sterile scalpel by practicing two symmetrical cuts from the sternum toward the diaphragm membrane along the costal margins, severing the costal cartilages. Mediastinum is exposed by pulling down the severed portion toward the bladder with sterile forceps, and a transversal incision of the sternum allows the access to the heart. After transection of the great vessels, the heart is removed maintaining the pericardial membrane intact and immediately submerged in a hermetic plastic box filled with iced cold cardioplegia solution (~300 ml) to wash the excess blood. Next, piglets' hearts are perfused as described in 3.2.

Adult pig hearts are obtained from farm pigs euthanized at the abattoir and immediately washed with iced cold cardioplegia. Upon excision, ventricles are immediately surgically separated from the rest of the tissue by cutting along the left anterior descending artery and the posterior descending artery and transported in a hermetic plastic box containing 500–600 ml of ice cold cardioplegia solution.

In both cases, the transportation is performed in an insulated polystyrene box filled with cooler packs.

### Heart Perfusion

**Figure 1:**
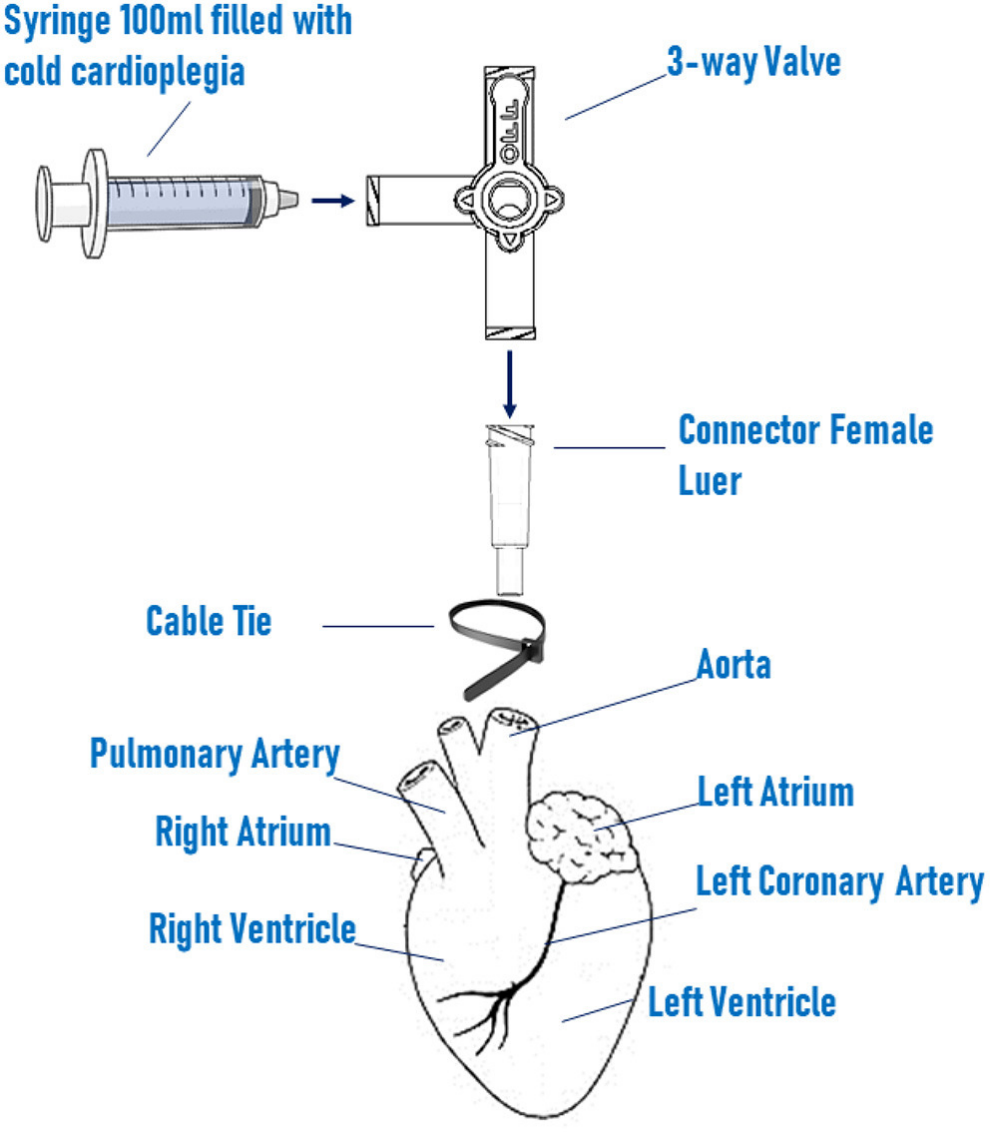
Schematic setting of heart perfusion system. Heart is perfused with cold cardioplegia *via* a 100 ml sterile syringe connected to a the 3-way valve. The valve is screw to a female luer connector, which is inserted in the ascending aorta and fixed in position by a nylon cable tie.

**Figure 2:**
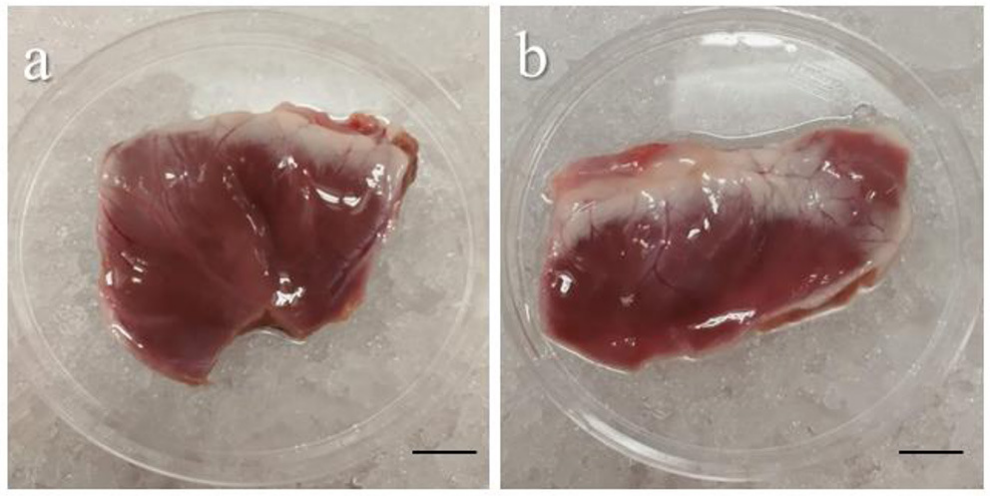
Representative images of piglet's ventricles after excision. Images showing the left **(a)** and the right **(b)** ventricles carefully removed from a piglet heart after heart perfusion. Freshly dissected tissue can be stored for up to 1 h in cold cardioplegia without affecting the epicardial viability. Scale bar, 1 cm. Source: Maselli et al. (23).

- Critical step: to ensure maximum/maximal survival of tissues, heart perfusion must be performed within few minutes from the excision.
- Screw a polycarbonate connector to one end of a 3-way valve, insert the luer in the aorta, and block it in position with a nylon cable tie (Figure 1).
- Fill a 100 ml sterile syringe with cold cardioplegia and connect at the 3-way valve, carefully pushing the cardioplegia though the valve. Tip: to avoid the formation of bubbles, turn the valve to direct the flow through the open end and flush the bubbles out before commencing the heart perfusion.
- Slowly inject the cardioplegia into the heart (c.a. 10–20 ml/min). Tip: successful perfusion can be assessed by monitoring the displacement of the blood by the colorless cardioplegia within the coronary arteries.
- After perfusion, the pericardial membrane is opened and the epicardium exposed.
- The ventricles are then removed and washed from any residual blood with cardioplegia and are ready for transportation (Figure 2). Important tip: for best results limit transportation time to a maximum of 1 h.

### Tissue Embedding and Slicing

Tip: Upon arrival in the laboratory, the solution temperature must be measured to ensure the preservation of the cold environment between 3 and 6°C.

NOTE: the equipment must be set up as described below before sample arrival:

#### Vibratome Setup

Clean the vibratome with 70% ethanol and distilled water and mount the blade (Wilkinson sword). It is essential to check the optimum positioning of the blade using Leica's VibroCheck, which minimizes the vertical vibration during cutting avoiding damage to the tissue. Set the cutting amplitude at 1.5 mm and adjust z-axis deflection of the blade, following the manufacturer instructions, at values comprised between −0.2 and 0.2 μm. Once the blade alignment is optimal, the vibratome bath can be mounted. The outer part of the bath is filled with ice, the inner part is filled to the top with cold NT solution (4°C). The solution is bubbled with 99.5% oxygen for at least 30 min before starting to cut.

#### Preparation of Tissue Dissecting and Embedding Equipment

**Figure 3:**
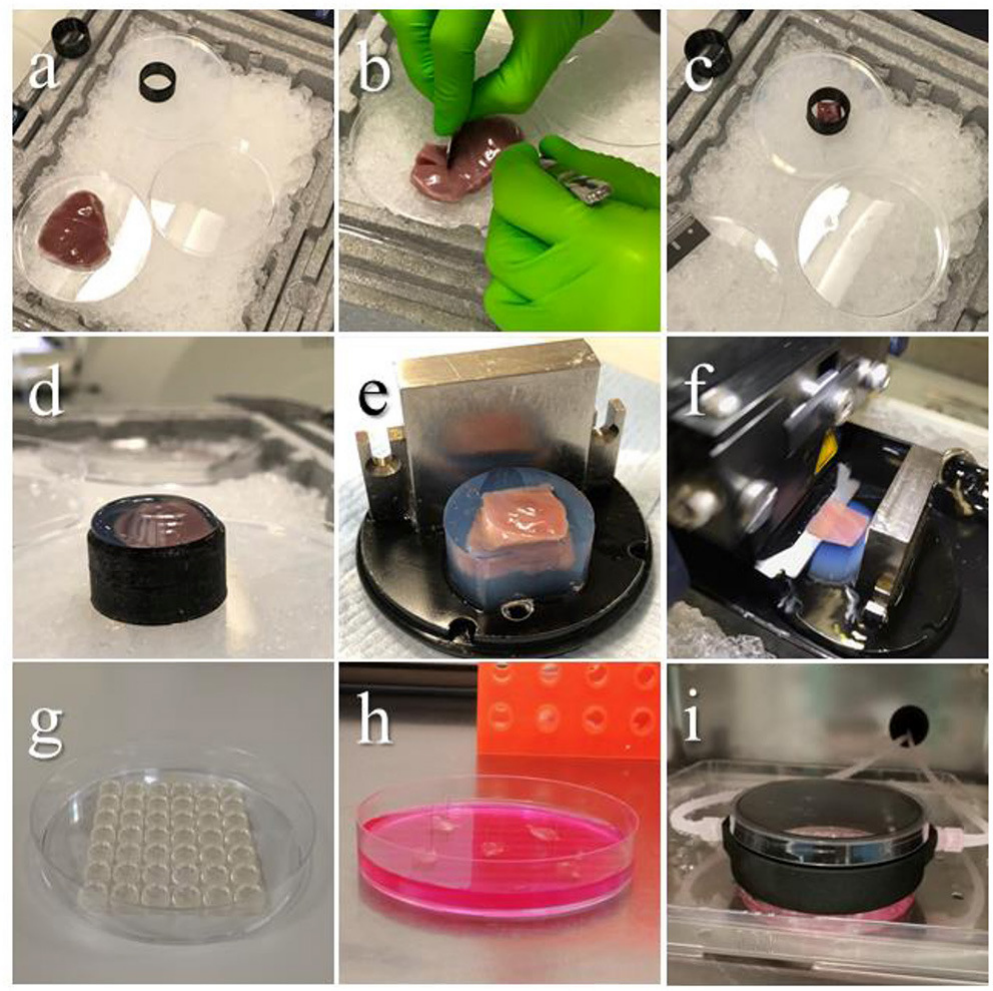
Embedding procedure and slicing. Images showing the steps of tissue preparation for cutting epicardial slices. **(a)** The ventricles are isolated avoiding any direct handling of the epicardium and placed on ice throughout the procedure. **(b)** Tissue blocks are dissected by making an incision through the ventricular wall with a single edge steel blade and **(c)** placed on cold agarose inside a 3D printed plastic ring, the epicardium facing down. **(d)** The plastic ring is filled with low melting agarose. **(e)** Once solidified, the block is removed from the ring, squared and mounted on the holder using cyanoacrylate glue. **(f)** Epicardial slices are cut using a vibratome, with the blade speed at 0.03 mm/s. **(g–i)** Epicardial slices are pinned to pillared Petri dishes, and either cultured in static conditions changing medium every 24 h, or connected to BioFlo120^Ⓡ^ benchtop bioprocessor through a custom 3D-printed insert, enabling flow culture and control of the culture conditions. Source: Maselli et al. (23).

For the tissue dissection and embedding area the following material is needed: a polystyrene box full of ice, a petri dish and the agarose cushion already prepared, organized as in Figure 3a. Tip: to avoid delays, set up the dissection area in the vicinity of a water bath and a fridge.

- To prepare tissue embedding solution, dissolve 5% low melting agarose in NT solution (w/v). Heat the mixture and stir it until the agarose has completely melted. Leave the solution to cool down in a water bath set at 37°C.
- Tissue embedding is performed on a cushion of 2% (w/v) cold agarose prepared in NT solution. To prepare the agarose cushion, dissolve 2% (w/v) agarose in NT solution, and after heating, pour the melted agarose solution into a petri dish lid and let solidified at room temperature.
- Additional required equipment are single edge steel blades, anatomical forceps, 3D printed plastic ring (Supplementary File 1), plastic Pasteur pipettes and cyanoacrylate glue.

#### Preparation of Recovery Bath

The recovery bath for the epicardial slices should be prepared in advance by placing 6-well dishes with central holes in a large plastic box filled with recovery solution at room temperature and bubbled with 99.5% oxygen. In each well of the 6-well plate, place a cell culture insert.

Once the low melting agarose solution is equilibrated at 37°C, the dissecting area is prepared and the agarose cushion is set, it is possible to start cutting the epicardial slices.

- Place the ventricle on a petri dish on ice (epicardium up), dissect out an 8 × 8 mm tissue block by making incisions through the full thickness of the ventricular wall with a single edge steel blade (Figure 3b).
- Lightly blot the surfaces of the tissue block, avoiding touching the epicardium, to remove the excess of solution.
- Gently place the tissue block on the agarose cushion with the epicardium facing the cushion and make the whole surface adhere to the agarose to flatten the cutting surface. **Critical step**: if the epicardial surface does not adhere completely to the agarose surface the viability of the myocardial side of the slice will be compromised.
- Place the 3D printed plastic ring to surround the tissue (Figure 3c) and use a Pasteur pipette to transfer the low melting agarose solution into the plastic ring (5–8 ml). Continue adding until all the tissue is covered and the solution is over the edge of the ring (Figure 3d).
- Once solidified, remove the block from the cushion and cut off the excess of the agarose along the ring top.
- Carefully remove the block from the ring and square one side of the agarose cylinder to obtain a flat surface and orientate the tissue on the specimen holder facilitating the alignment of the blade (Figure 3e).
- Apply a drop of cyanoacrylate glue on the specimen holder and place on it the endocardial side of the embedded tissue block, waiting until the block is firmly glued on the holder, then mount on the vibratome.
- Position the vibratome' blade on top of the block and set this position as starting point, then set the cutting thickness to 400–500μm and start slicing (Figure 3f). Critical step: this step is critical to obtain thin and homogenous tissue slices, and to minimize the difference between the setup value and the final thickness. The critical point is to set the starting point as close as possible to the block surface. Tip: the blade has to make contact with the block surface to improve precision of the positioning. If possible, ensure this is done on the agarose surrounding the tissue. Avoid sliding the blade over the epicardial layer.
- While slicing, advance the blade at 0.03 mm/s. Once the blade has completely cut through the tissue, reaching the agarose on the posterior part of the block, stop the blade and return it to the starting position. Carefully collect the slices with the forceps by holding it from the corner, removing the excess agarose, and transfer in one of the cell culture inserts in the recovery bath. Let the slices recover at least 30 min to up to 4–5 h in the recovery bath. Slices might start to curl, but this will not affect viability, their culture or the outcome of the experiments.
- Multiple epicardial slices can be obtained from different blocks. After the removal of the epicardial slice, myocardial slices can be cut from the same block.

### Culture of Epicardial/Myocardial Slices

Once recovered, slices can be cultured epicardium-up in Petri dishes modified by creating a PDMS pillar arrays at the bottom. Culture system is a modification of the previously published air/liquid interface method (24). The pillars are used to pin the slices, positioning an entomologist pin at each of the four corners of slice. This helps maintaining flatness and maximizes the contact between the tissue and the culture medium. Here is described an efficient and inexpensive method to produce the pillar array.

#### PDMS Pillar Array Fabrication

The PDMS is prepared by mixing the liquid silicone base with the curing agent (10:1, w/w) and carefully mixing avoiding formations of bubbles. To create the pillar array, pour 25 ml of the elastomer mixture in a shallow container (such as the lid of a multi-well dish) and press a 1,000 ml tip rack on top, ensuring that the solution fills the holes. Any master mold with spaced holes 5–8 millimeters deep would be appropriate. Polymer is cross-linked at 65°C for 3 h. Once solidified, the array can be cut to fit in the Petri dish as in Figure 3g and autoclaved in a sterilization pouch. The same array can be washed, autoclaved and re-used multiple times.

#### Culture System Setting

Epicardial slices can be cultured efficiently in the pillared Petri dish, changing medium every 24 hours (Figure 3h). However, here we describe a tunable culture method which enables a fine and real-time control/adjustment of the culture parameters, such as: pH, dissolved O_2_ and CO_2_, and medium exchange rate. We implemented this culture method using the BioFlo120® benchtop bioprocessor which monitors and regulates these parameters in a reservoir and pumps the medium from the reservoir to the culture dish via a custom 3D-printed insert, as shown in Figure 3i. The insert, made of thermo-resistant plastic (PA12 Nylon), was printed according to the design in the Supplementary File 2. Before culture, the reservoir is filled with 300 ml of medium (Medium 199 + 1X ITS Liquid Media Supplement + 1% Penicillin/Streptomycin Penicillin-Streptomycin + 10 mM of BDM) and parameters are set at pH 7.4, 21% oxygen level and medium flow of 4 ml/min, following to the manufacturer's instructions. The 3D insert must be autoclaved with input and output valves closed with caps before every use.

The following steps must be carried out in a sterile laminar flow hood:

- Transfer the slices from the recovery bath to the hood using a clean multi-well plate. Then, under the hood, move the slices in a new sterile multi-well plate and wash the slices by gently adding 0.22 μm filtered NT solution supplemented with 2% Penicillin-Streptomycin.
- Using sterile forceps, put the slice on top of the pillar array, and stick each corner on a pillar using entomologist pins, previously sterilized with 70% ethanol. Add 25 ml of medium into the dish.
- Place the 3D printed insert in between the plate and the lid and move the plate to the incubator, before removing the caps and connecting the input and output tubes.
- Activate the medium flow and incubate at 37°C in 5% CO_2_ for up to 72 h.

### Immunohistochemical Analysis

The slice viability, morphology and cellular composition can be assessed by histological analysis. Following is a protocol to obtain cross sections of the slices and perform immunohistochemical analysis.

- Fix the slices in 4% PFA (Paraformaldehyde, Santa Cruz Biotechnology) overnight at 4°C.
- Wash with phosphate buffer saline (PBS) and incubate overnight in 30% sucrose (Sigma-Aldrich) solution in PBS (w/v).
- To embed slices, lay them flat in a mold, cover in OCT Compound (Agar scientific) and snap freeze in isopentane cooled in liquid nitrogen.
- Cut 5 μm thick longitudinal cryosections (Hyrax C25 Cryostat, Zeiss), perpendicular to the epicardial surface, and collect them on SuperFrost slides (Menzel-Glaser, Germany). Slides can be stored at −20°C for up to 3 months.
- Hematoxylin/eosin staining can be performed for histological investigations. Results in **Figure 5** were obtained using Hematoxylin and Eosin Stain Kit (Vector Laboratories, H-3502) following the manufacturer's instructions.
- Antibody staining conditions must be optimized by the operator; however, below are some suggestions to improve the results of desired immunohistochemical assays:

∘ Antigen retrieval is performed using citrate buffer (0.1 M Citric Acid, pH 6.0) for 5 min, 3 times in microwave at 759 W.
∘ For nuclear antigens, permeabilize at room temperature for 30 min with 0.1% Triton-X 100 (v/v) in PBS (Sigma-Aldrich).
∘ Block unspecific antigen binding using 20% (v/v) Goat serum (Sigma-Aldrich) in PBS for 1 h.
∘ Incubate with primary antibody at 4°C overnight. Results in **Figure 5** were obtained using the following antibodies and concentrations: WT1 1:50 (ab89901), CD31 1:100 (ab28364) both from Abcam; Mesothelin 1:100 (NB110-85538) from Novus Biologicals; α-Actinin (Sarcomeric) 1:800 (A7732), α-smooth muscle actin (α-SMA) 1:400 (A5228) both from Sigma-Aldrich; Connexin 43 1:300 (71-0700), Vimentin 1:100 (MA5-11883) both from Thermo Fisher Scientific, followed by the appropriate Goat anti-Mouse and/or Goat anti-Rabbit Alexa Fluor (Thermo Fisher Scientific) secondary antibody diluted 1:200 for 1 h at 37°C.
∘ Stain the nuclei with DAPI 1 ug/ml (4′,6-Diamidine-2′-phenylindole dihydrochloride, Sigma-Aldrich) for 10 min at room temperature.
∘ To help to reduce tissue autofluorescence, incubate 30 min with 0.1% (w/v) Sudan Black (Sudan Black B, Santa Cruz Biotechnology) solution in 70% ethanol (w/v).

Whole mount histological analysis can be performed after tissue fixation (4% PFA, overnight at 4°C) and decoloration with CUBIC (Clear, Unobstructed Brain/body Imaging cocktails and Computational analysis) solution made with 25% (w/v) of urea (Sigma-Aldrich), 25% (w/v) of N,N,N,N-tetrakis(2-hydroxypropyl)ethylenediamine (Alfa Aesar) and 15% (w/v) Triton X-100 (Sigma-Aldrich) in water (30, 31). Briefly, whole slice decolouration is carried out for 6 days at room temperature on a slow shaker, changing solution every 2 days. Slices are then blocked with 20% (v/v) Goat serum (Sigma-Aldrich) in PBS for 24 h. Primary antibody incubation is performed at room temperature for 48 h (as in **Figure 6**, WT1 1:50 from Abcam and Mesothelin 1:100, from Novus Biologicals), followed by washing for 6 h in PBS. Finally, incubate with the appropriate Goat anti-Mouse and/or Goat anti-Rabbit Alexa Fluor secondary antibody at room temperature for 48 h and wash in PBS for 24 h.

### Nanoneedle-Mediated Tracking of Epicardial Cells

Nanoneedles are an innovative nanoscale transfection tool (32–34) capable of transferring a variety of payloads, including nucleic acids and fluorescent quantum-dots, into the cytoplasm of cells without affecting their viability. Porous silicon nanoneedles are arranged vertically in an array with 2 μm spacing on an 8 × 8 mm chip; each needle is tailored to be 5 μm long with tips <50 nm and 50–70% porosity. The high porosity and the sharp tip geometry combine a high loading capacity with an efficient intracellular delivery (32, 35). Using this platform, we can deliver plasmids expressing reporter genes or cell impermeable dyes to the cells residing on the surface of the slice, within the epicardium.

Here we describe a protocol to deliver pcDNA3.1-GFP plasmid to the epicardial cells situated on the superficial layer of our slices using nanoneedles. Tagging the cells will enable fate mapping studies in large animals' adult tissues *ex vivo*. Similar approaches can be devised to transfer other genes of interest exclusively to the epicardium, to test their function.

- Nanoneedles are fabricated as previously reported (35) and treated with oxygen plasma for 10 min (0.4 mbar, 200 Watt), followed by coating 1 h with poly-L-lysine solution 0.1% (w/v, Sigma-Aldrich) in H_2_O at room temperature. Nanoneedles are then washed for 30 s with 2N HCl (Sigma-Aldrich) followed by two washes for 30 s with distilled water.
- Plasmid loading is performed by incubating the chip surface for 15 min at room temperature with 20 μl of 1 μg/ml plasmid solution in H_2_O, followed by a quick wash with sterile PBS.
- Nanoneedle chips are then applied on epicardial surface of the tissue slices for 30 min and then carefully removed.
- GFP expression is visualized with Nikon Eclipse Ts2 inverted microscope after 48 h of culture (as described above).

## (Anticipated) Results

### Viability of Slices

We used different assays to test the viability of the epicardial/myocardial slices. Epicardial cells metabolic activity/viability can be evaluated by Calcein acetoxymethyl (Calcein AM) (Thermo-Fisher) live staining, while for the myocardium utilized multi-electrode array (MEA) (MEA1060, Multi Channel Systems, Reutlingen, Germany) which allow non-invasive multifocal recording of extracellular field potential.

#### Live Staining

**Figure 4:**
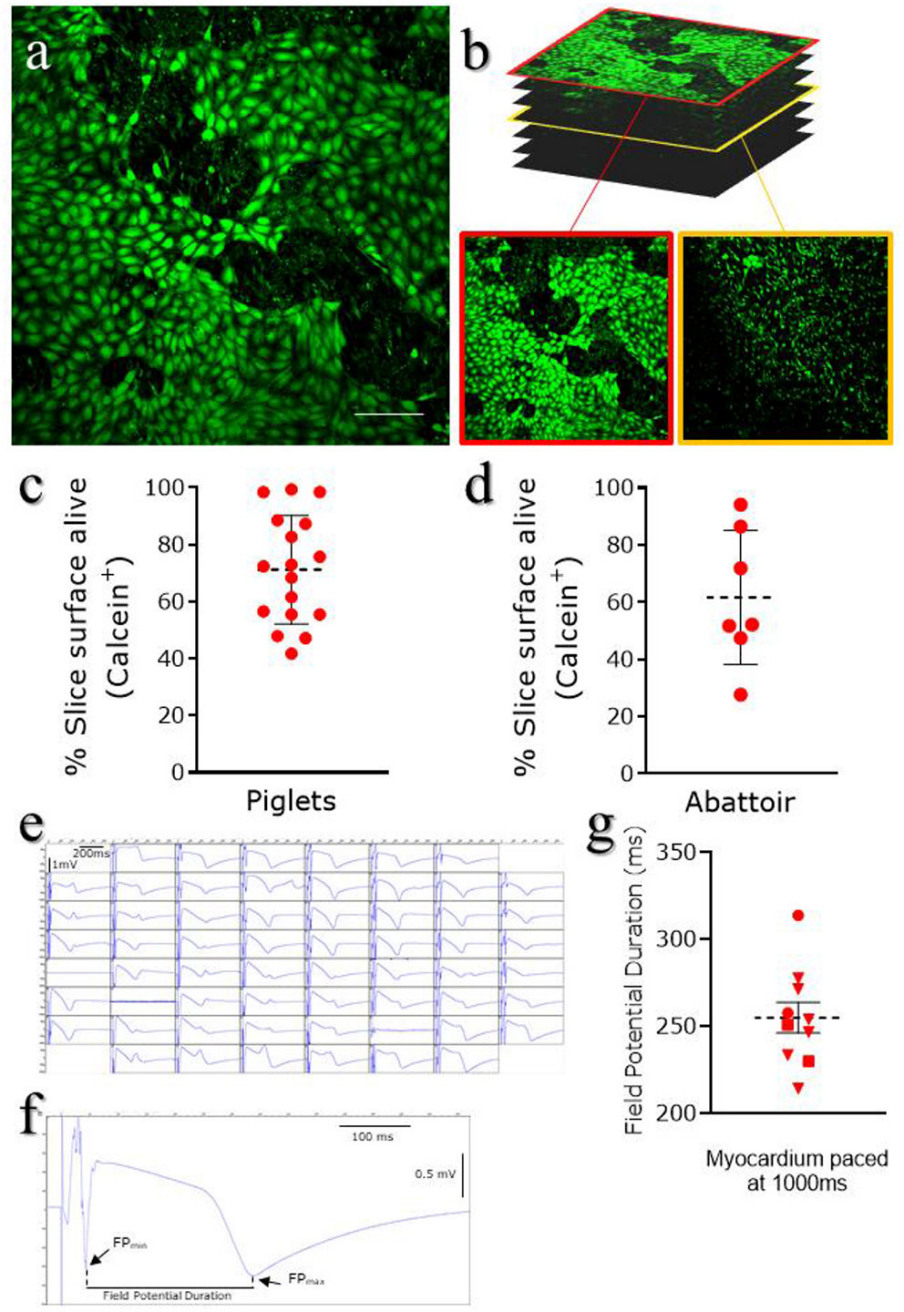
Epicardial slices viability. Viability was assessed using Calcein AM live staining. Live cells are stained green. **(a)** Representative image of live staining of an epicardial slice imaged using confocal microscopy. Scale bar, 100 μm. **(b)** Representative images of Z-stack confocal acquisition of live/dead staining of epicardial slice, showing epicardial cells monolayer on the surface of the slice (red outline) and underlying fibroblasts/myocardium (yellow outline). **(c)** Quantification of alive area of the surface of piglet slices (*Mean and SEM*: 71.21 ± 4.61%; N of slices = 17/ N of Pigs = 10) and **(d)** abattoir-derived slices (*Mean and SEM*: 61.65 ± 8.88%; N of slices = 7/ N of Pigs = 4). **(e)** Representative field potential traces recorded from cardiomyocytes within the epicardial slices and **(f,g)** field potential duration, measured as the time between the depolarisation peak (FP_MIN_) and the repolarization peak (FP_MAX_) (*Mean and SEM* was 254.87 ± 8.86 ms; N of slices = 10/ N of Pigs = 3).

Calcein AM (Thermo Fisher Scientific, C1430) is a cell-permeant compound, converted in green fluorescent calcein by intracellular esterases. Microscopic imaging of the tissue stained with Calcein AM provides both functional and morphological information: the fluorescent conversion indicates the metabolic activity of cells and highlight the typical cobblestone morphology of the squamous epithelium (Figure 4a). Sporadic interruptions of the epicardial monolayer exposes the underlaying connective tissue and fibroblasts (Figure 4b). Morphological evaluation of the slices is fundamental to assess the outcome of the slice cutting protocol, with successful preparations presenting brightly fluorescent epicardium and extensive epicardial coverage. Briefly, slices are stained in NT solution supplemented with 10 μM Calcein AM cell-permeant dye at room temperature for 45 min under continuous shaking. Slices are then washed twice for 5 min with NT solution to remove all the Calcein AM residues and kept on ice until imaging by confocal microscopy. Z-stack confocal images are captured from random fields of the slice surface, using a 10 × objective on a Nikon Eclipse Ti A1-A confocal laser scanning microscope and max projection images are quantified to estimate the percentage of live surface. We found that on average 71.21 ± 4.61% of the surface of epicardial slices from piglets (Figure 4c) and 61.65 ± 8.88% of the abattoir-derived slices were viable (Figure 4d).

#### Multi-Electrode Array Measurements

MEA system can be utilized to electrically stimulate the myocardial fibers present on the underside of the slices, evaluating the electrophysiology of the tissue (29). Data were acquired using a dish which contains 60-microelectrode arranged in an 8 × 8 matrix and an inter-electrode distance of 700 μm, providing a recording area of 4.9 × 4.9 mm^2^. Slices are positioned in the center of the MEA dish, over the recording area, and submerged in oxygenated NT solution for recording at 37°C (see info in Table 1). Myocardial fibers within the epicardial slices exhibit viable field potential traces in the large majority of microelectrodes within the first 5 h following cutting, when elicited by 1 Hz stimulation (Figure 4e). This result demonstrates full functionality and the maintenance of electrical connections between cardiomyocytes within the cardiac slices. The measurement of the field potential duration (Figure 4f), which is defined as the time between the depolarisation peak (FP_MIN_) and the repolarisation peak (FP_MAX_), estimates the action potential duration at 90% of repolarisation (APD90) (36). Our analysis, performed with Clampfit software (Axon Instruments, USA), indicate an average baseline of field potential duration of 254.87 ± 8.86 ms, which is in line with the value of field potential duration measured in cardiac slices of comparable size mammals (29) (Figure 4g).

### Morphological Analysis

The tissue architecture of the slices can be investigated using different techniques. Here we show the results from the hematoxylin and eosin coloration and immunohistochemistry, following tissue sectioning, and the whole mount staining on decolorated epicardial/myocardial slices.

#### Tissue Sections

**Figure 5:**
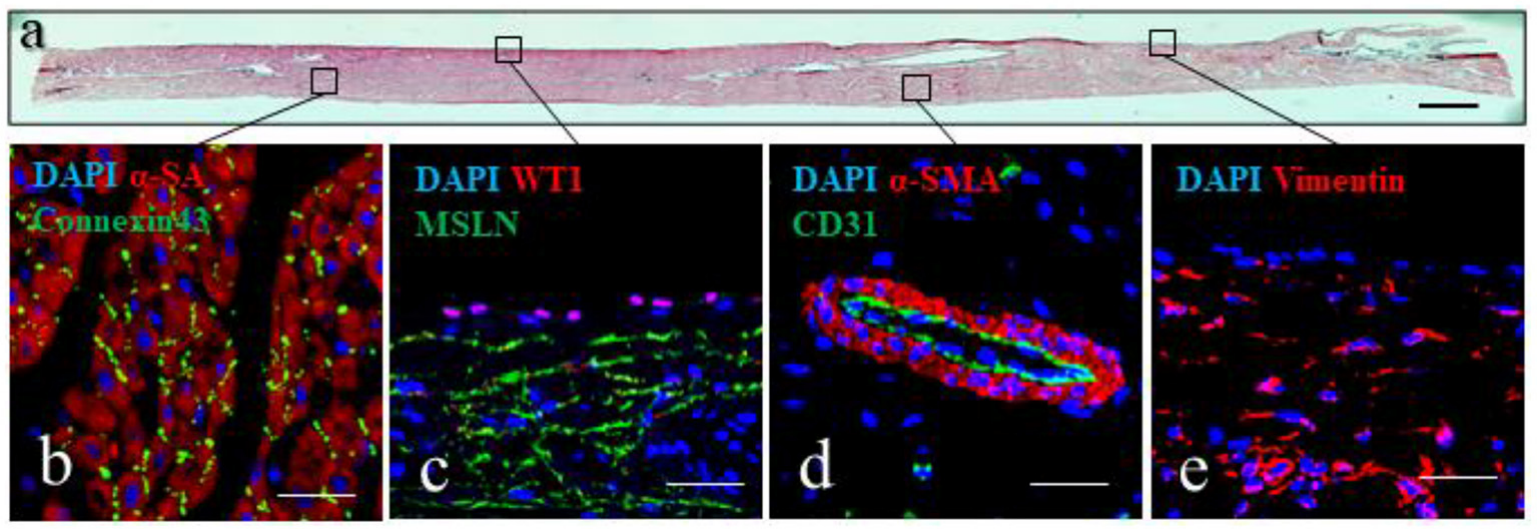
Epicardial slices structure analysis. **(a)** Tissue architecture evaluation of epicardial slices performed using hematoxylin and eosin coloration. Scale bar, 500 μm. **(b–e)** Immunohistochemical staining and confocal microscopy of epicardial slices. **(b)** Cardiomyocytes are stained with α-sarcomeric actin and the intercalated disk is stained with connexin-43 antibody. **(c)** Epicardial cells highlighted with WT1 and mesenchymal derived cells stained with MSLN. **(d)** Microvasculature is stained with α- smooth muscle actin and the endothelial marker CD31. **(e)** Mesenchymal cells are stained with vimentin. Nuclei are labeled with DAPI. Scale bars, 50 μm.

Histochemical analysis of the epicardial/myocardial slices performed with hematoxylin and eosin (Figure 5a) shows the orientation of myocardial fibers running parallel to the epicardial layer. Large coronary vessels and low levels of adipose tissue and connective tissue are also visible. High magnification images show the results obtainable from immunofluorescence staining of the slices (Figures 5b–e). Sarcomeric actin (α-SA) and connexin43 staining indicate the preservation of the myocardial tissue architecture, with sarcomeric structures and intact gap junctions (Figure 5b). Retention of intact epicardial monolayer is assessed by the expression of the epicardial progenitor transcription factor WT1 and the membrane marker mesothelin (MSLN) (Figure 5c). Preserved vascular tree is evidenced by the staining of capillaries, venules and arterioles with endothelial cell marker CD31, in some cases enveloped by α-SMA+ perivascular cells (Figure 5d). Connective tissue is identified by the expression of vimentin, which is typically highly expressed by mesenchymal/fibroblast cells (Figure 5e).

#### Slices Decoloration

**Figure 6:**
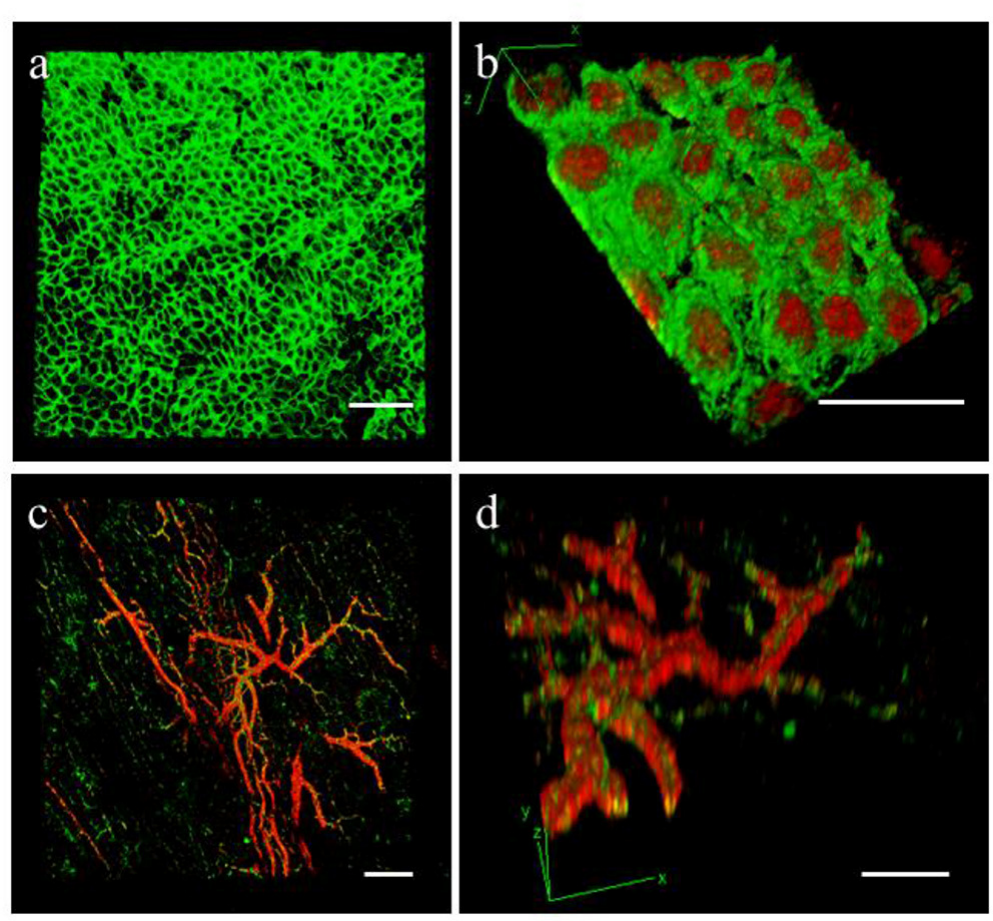
Epicardial slices decolouration. Immunohistochemical staining and confocal analysis 3D reconstruction of decolorized epicardial slices obtained from piglet hearts, showing **(a)** low magnification image of the epicardial layer stained with MSLN (green) and **(b)** high magnification image showing the epicardial monolayer stained with MSLN (green) and WT1 (red) from the bottom. Low magnification **(c)** and detail view **(d)** of the vasculature present into the epicardial slice a stained with smooth muscle marker α-SMA (red) and the endothelial marker CD31 (green). Scale bars, 50 μm.

We used the optical clearing method to visualize the expression of WT1 and MSLN in the full thickness of the epicardial monolayer (Figures 6a,b). We also obtained a comprehensive 3D map of the macro- and micro-vasculature within the slices by CD31 and α-SMA labeling (Figures 6c,d).

### Culture

**Figure 7:**
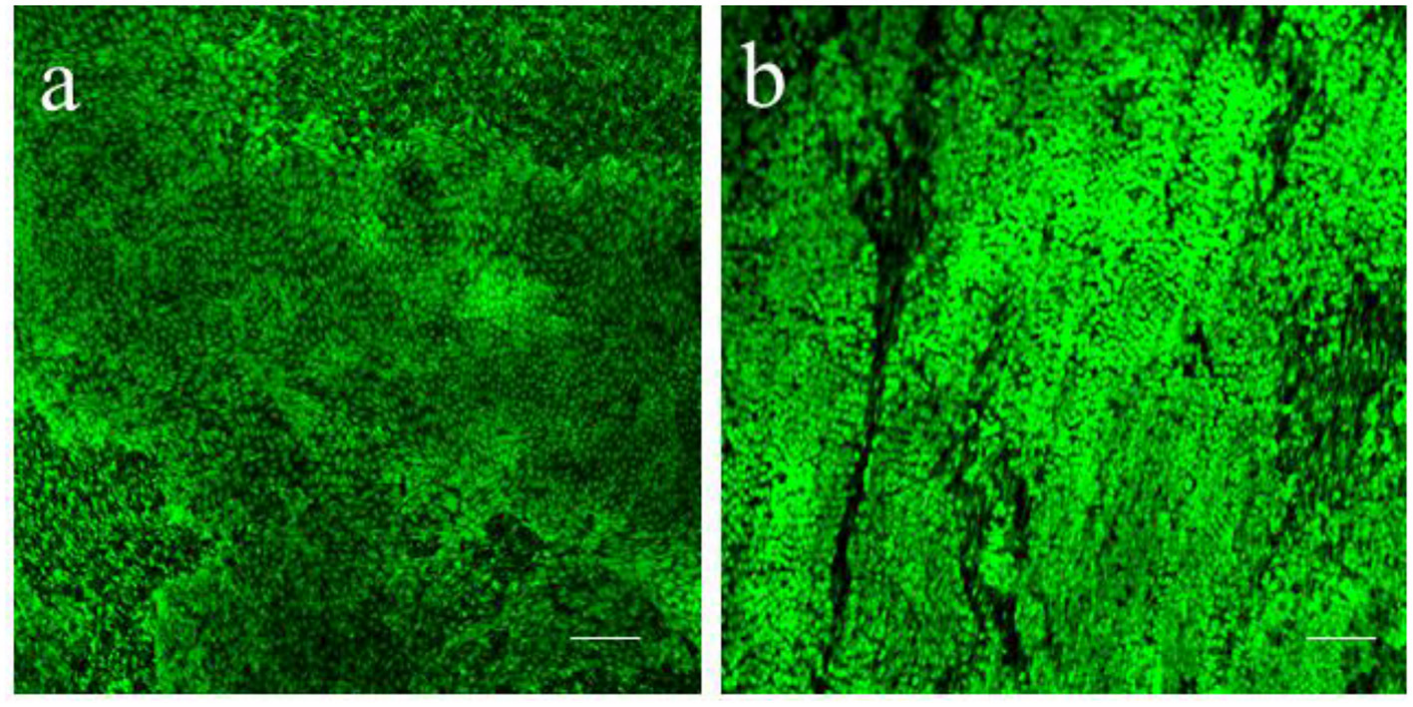
Epicardial slices viability in culture. Calcein AM live staining performed on **(a)** piglet and **(b)** adult swine-derived slices, after 72 h of culture. Both tissues display large portion of living epicardial cells and the retention of the typical cobblestone morphology. Scale bars, 100 μm.

To keep the epicardial/myocardial slices alive, we developed a culture system which builds on the air-liquid interface protocol previously reported for the culture of myocardial slices (37). Our method allows tissue-medium contact at both epicardial and myocardial side, which is proven to be positive for the performance of the slices in culture (38). Notably, *en face* staining showed high epicardial cells viability and the retention of the typical cobblestone morphology in both piglets and adult swine hearts after 72 h of culture (Figures 7a,b).

### Nanoinjection of Plasmid

**Figure 8:**
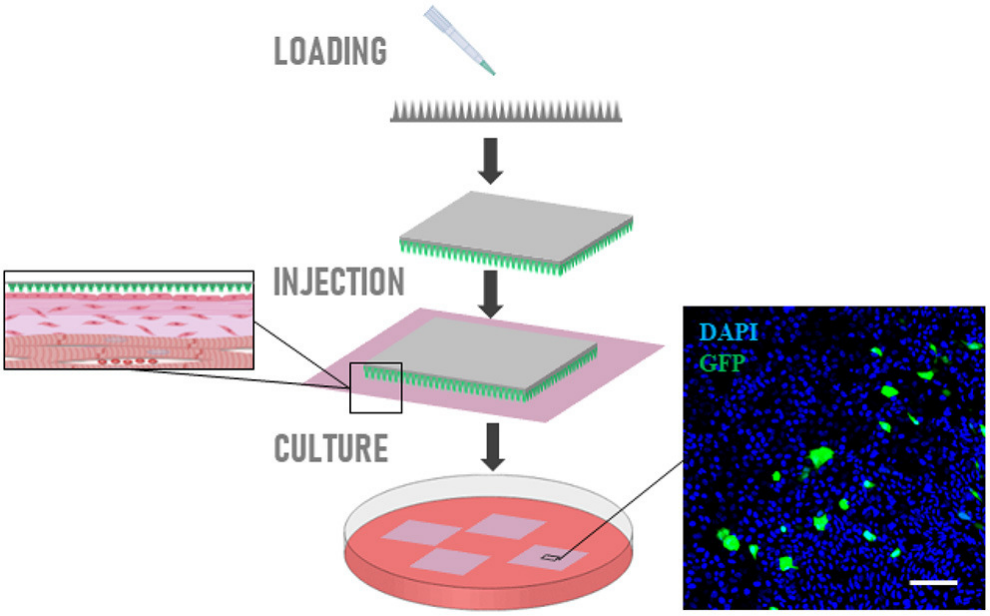
Nanoinjection of plasmid. Schematic representation of nanoneedles-mediated delivery of GFP-carrying plasmid on epicardial cells. Confocal imaging indicated expression of GFP 48 h after nanoinjection in the epicardium of cultured slices. Scale bars, 50 μm.

GFP-carrying plasmid can be efficiently loaded on nanoneedles and delivered to the epicardial cells on the slice surface. Confocal imaging indicated expression of GFP 48 h after nanoinjection in the epicardium of cultured slices (Figure 8).

## Troubleshooting Guide

In this section, we address the most common issues related to the preparation of epicardial/myocardial slices and suggest some possible solutions.

### Tissue Block Shows Decoloration at the Edges After Embedding

- Low melting agarose solution is too warm:

∘ Check the temperature of the solution and ensure the water bath is set at 37°C.

### Blade Is Unable Cut the Tissue

- Blade is damaged or not mounted correctly:

∘ Remove and replace the blade (check the z-axis deflection with VibroCheck).

- Tissue block is dis-homogeneous due to adipose or connective tissue:

∘ Epicardial/myocardial slice integrity and viability are compromised, remove the tissue block and proceed with another embedding. This issue is more frequent in proximity of big vessels, try to avoid these areas when preparing the tissue block.

### Epicardium Slice Is Not Flat

- Embedding agarose block is not flat or not well glued on to the specimen holder:

∘ Myocardial side viability is compromised, this affects the viability of the whole slice in long-term culture. Remove the tissue block and proceed with another embedding. Be careful during the embedding and when removing the block from the plastic ring not to damage the agarose or press in correspondence of the tissue as this might cause the epicardium to bulge.

### Epicardial Slice Appear to Be Thick

- Wrong position of the blade:

∘ This can affect the viability of the slice. Remove the tissue block and proceed with another embedding. Set the vibratome's z-axis cutting blade starting position at the top edge of tissue block, ensure the blade is in contact with tissue/agarose when you set it.

### Epicardial Slices Curl-Up Quickly in Recovery Solution

- Solutions were made incorrectly:

∘ Slice viability can be compromised. Prepare the solution using ultrapure sterile water, adjust the pH at the exact working temperature of the solution, check the osmolarity to be in between 290 and 320 mOsm/L.

### Poor Survival in Culture

- Lack of air-liquid interface:

∘ The culture medium must skim the edges of the slice while leaving the epicardium partially exposed to the atmosphere, ensuring liquid interchange by capillarity. The correct amount of medium and the pump's speed need to be calibrated by the users.

## Advantages and Limitations

The increasing interest in the epicardium's regenerative capacity stimulated the development of numerous protocols to obtain cell systems for *in vitro* studies. Epicardial cells can be isolated by enzymatic digestion, by exploiting their ability to migrate from epicardial explant or by differentiation from stem cell sources (39–41). In terms of isolation strategy, epicardial outgrowth implies a degree of cell activation, while marker expression-based isolation and differentiation is complicated by the heterogeneity of the epicardial population, and the ectopic expression of markers (e.g., WT1) (23, 42, 43). The resulting cell population might therefore be contaminated by unwanted cell types and/or present different characteristics as compared to the *in vivo* counterpart. Furthermore, these primary cell lines have a tendency to undergo spontaneous epithelial-to-mesenchymal transition in culture, limiting their suitability for this type of studies (40). While primary cell studies have undoubtedly yielded important findings, epicardial slices represent a powerful tool in the investigation of the epicardium. Indeed, epicardial cells within the slices are embedded in their natural microenvironment, reducing spontaneous differentiation while at the same time enabling studies of the interaction between different cell populations within the cardiac tissue (23). In addition, epicardial/myocardial slices present significant advantages in respect to the currently available models: (i) Organotypic slices can be easily obtained and cultured *in vitro* while preserving the complexity of the tissue *in vivo*, maintaining tissue/cell morphology and the connections within cells and with the extracellular matrix. This integrated system allows to measure the effect of a treatment on multiple cell targets at once, providing information on epicardial, myocardial and non-contractile cells of the heart as well as the extracellular matrix. (ii) Slice preparation is cost effective as compared to large animal experiments. Moreover, from a single heart is possible to obtain up to 15–20 slices, increasing the number of samples/replicates and reducing the intra-assay variability. (iii) Our *ex vivo* model is accessible to the majority of the laboratories, as epicardial slices can be obtained from abattoir-derived hearts. (iv) Additionally, the wider application of epicardial/myocardial slices will encourage the reduction of the number of animals used in research, replacing in part the use of rodents, large animals and zebrafish. At the same time, given the close similarities between porcine and human heart, results obtained on the slices will be clinically relevant.

While presenting some obvious advantages, slices also have some limitations: (i) The procedure is time-consuming and requires training and some preparatory experiments to ensure the success of the procedure. However, this limitation has to be weighed against the equally complex and time-consuming and ethically challenging training for *in vivo* work. (ii) Short-term viability in culture, as compared to classic *in vitro* models. Slices can be cultured up to 72 h, that could represent a limitation when mimicking chronic disease onset. Extended culture might be possible, although we expect some loss of viability in the myocardial compartment, and further studies are required to increase the lifespan of the slices. (iii) Lack of the inflammatory cells in the culture environment. Due the increasing interest for the role of the immune system in cardiovascular diseases, the integration of inflammatory cells in slices culture is one of the main targets for the development of this model. Immune cells and inflammatory stimuli can be added to the culture media as in a normal *in vitro* experiment, helping to identify specific mediators. (iv) Difficulties to distinguish the epicardial contribution due to the complexity of the tissue. The role of the epicardium can be assessed both by tracking the cells using methods such as the nanoinjection described here, or by comparison with myocardial slices obtained from the same block.

## Data Availability Statement

The raw data supporting the conclusions of this article will be made available by the authors, without undue reservation.

## Ethics Statement

This study was reviewed and approved by the Animal Welfare and Ethical Review Board (AWERB) of The Pirbright Institute.

## Author Contributions

DMas conceived and planned the experiments and analyzed the data. RM, RJ, DMar, and VC provided key experimental expertise. DMas and PCamp wrote the manuscript and prepared the figures with support of CC and PCame. PCamp, PCame, and CC conceived the original idea and supervised the project. All authors contributed to the article and approved the submitted version.

## Funding

This work PCamp and DMas were supported by the National Centre for the Replacement, Refinement & Reduction of Animals in Research (grant numbers: NC/R001006/1 and NC/T001216/1). RM is supported by the Doctoral College studentship award (University of Surrey) and CC, DMar, and VC by the European Research Council (grant reference: StG EnBioN 759577). PCame and RJ acknowledge support from the British Heart Foundation (grant number: FS/17/33/32931), the Royal Society (RSG/R1/180198) and the HEIF Strategic Fund.

## Conflict of Interest

The authors declare that the research was conducted in the absence of any commercial or financial relationships that could be construed as a potential conflict of interest.

## Publisher's Note

All claims expressed in this article are solely those of the authors and do not necessarily represent those of their affiliated organizations, or those of the publisher, the editors and the reviewers. Any product that may be evaluated in this article, or claim that may be made by its manufacturer, is not guaranteed or endorsed by the publisher.
